# Disease Outcomes of COVID-19 in Diabetic and Hypertensive Patients During the Hospital Stay

**DOI:** 10.7759/cureus.46943

**Published:** 2023-10-13

**Authors:** Amina Abid, Arooj Umar, Samina Qamar

**Affiliations:** 1 Internal Medicine, Mayo Hospital Lahore, Lahore, PAK; 2 Internal Medicine, Shaukat Khanum Memorial Cancer Hospital and Research Centre, Lahore, PAK; 3 Internal Medicine, University Hospitals Coventry and Warwickshire NHS Trust, Coventry, GBR; 4 Pathology, King Edward Medical University, Lahore, PAK

**Keywords:** nrm, bipap, cpap, inflammatory markers, covid-19, diabetes, hypertension

## Abstract

Background

A single-stranded RNA genome-encapsulated virus known as severe acute respiratory syndrome coronavirus 2 is known to cause severe acute respiratory syndrome in humans. People with diabetes and hypertension are often more susceptible to developing coronavirus disease 2019 (COVID-19) and experience a disproportionately higher rate of morbidity and death compared to the general population. The COVID-19 pandemic has become an urgent worldwide issue. Therefore, the main goal of this study is to assess how diabetes and hypertension, both separately and together, affect clinical outcomes in terms of morbidity and mortality in patients hospitalized with COVID-19. This study aimed to evaluate the disease outcomes in hypertensive and diabetic patients hospitalized with COVID-19.

Methodology

This descriptive, cross-sectional study was conducted from June 2022 to November 2022. Using purposive selective sampling, a total of 90 known hypertensive and diabetic patients with COVID-19 aged 18-90 years admitted in COVID-19 isolation wards and intensive care units (ICUs) of Mayo Hospital Lahore were recruited in this study after obtaining informed consent and IRB approval from the Institutional Review Board of King Edward Medical University, Lahore. Patients who did not provide consent, patients whose positive polymerase chain reaction reports for COVID-19 were not available, pregnant females, and patients with other comorbidities were excluded from the study. Data were collected from the COVID-19 isolation medical wards and ICUs from patient charts containing age, the status of hypertension and diabetes, disease status, severity, and levels of inflammatory markers, i.e., D-dimers, serum lactate dehydrogenase (LDH), serum ferritin, C-reactive protein (CRP). Data were analyzed using SPSS version 23 (IBM Corp., Armonk, NY, USA). Quantitative variables such as age were presented as mean ± SD. Qualitative variables such as hypertension, diabetes, and levels of inflammatory markers were presented as frequency and percentages.

Results

In this study, 90 patients were included, with 51 (57%) females and 39 (43%) males, all of whom were either hypertensive, diabetic, or both. In total, 70 (78%) patients were admitted to ICUs and 20 (22%) to COVID-19 medical isolation wards. Among 70 ICU patients, 39 (43.3%) were on continuous positive airway pressure/bilevel positive airway pressure, seven (7.8%) were on ventilators, and 44 (48.8%) were on normal oxygen masks/non-rebreather masks with high-flow oxygen. Overall, 100% of the patients included in the study had raised levels of inflammatory markers, low lymphocyte count, and increased neutrophil count. In total, 84 (93%) patients had severely high and six (7%) patients had moderately high CRP levels. Moreover, 33 (36.7%) patients had severely high and 57 (63.3%) patients had moderately high D-dimer levels. Further, 25 (28%) patients had severely high, 26 (29%) patients had moderately high, and 39 (43.3%) patients had significantly raised levels of serum ferritin. In total, 21 (23%) patients had severely high, 37 (41%) had moderately high, and 32 (36%) had significantly raised levels of serum LDH. Among the 90 patients, 65 (73%) expired and 25 (27%) survived. Of the expired patients, 62 (95%) were admitted to ICUs, and three (5%) were admitted to wards.

Conclusions

Diabetes and hypertension are strong predictors of COVID-19 severity in terms of morbidity and mortality due to respiratory deterioration.

## Introduction

Severe acute respiratory syndrome coronavirus 2 (SARS‑CoV‑2) is a single-stranded RNA genomic-enveloped virus known to cause severe acute respiratory syndrome in humans. Individuals with hypertension and diabetes mellitus are usually more likely to be affected and experience relatively increased morbidity and mortality due to COVID-19 compared to the normal population because of multiple factors including nitric oxide deficiency which is more pronounced in diabetics and hypertensives leading to respiratory deterioration [1]. Hence, hypertension and diabetes are considered the most prevalent comorbidities in COVID-19 patients [2].

A study of 191 patients with these comorbidities reported a mortality risk of 2.85-fold for diabetes and 3.05-fold for hypertension [3]. Similarly, the prevalence of diabetes and hypertension was higher among patients hospitalized for COVID-19 in intensive care units (ICUs) or in those who died [4].

According to recent studies, hypertension and diabetes have been suggested as the most frequent comorbid factors in patients with COVID-19 and have been considered independent risk factors in disease outcomes, such as morbidity and mortality [5].

Hypertension has been reported to occur in two-thirds of diabetes patients [6]. Yet, few studies have addressed the individual effects of hypertension and diabetes or their combination on the risk of morbidity and mortality in COVID-19 to date.

The main purpose of this study is to evaluate the impact of hypertension and diabetes individually and in combination on clinical outcomes in terms of morbidity and mortality in patients hospitalized with COVID-19 with systemic review and regression analysis using several indicators such as the mode of admission (either ICU or wards), type of oxygen source (face masks, mon-rebreather masks (NRMs), continuous positive airway pressure (CPAP)/bilevel positive airway pressure (BIPAP), ventilators), and number of deaths.

## Materials and methods

Study overview

This descriptive, cross-sectional study was conducted from June 2022 to November 2022. A total of 90 known hypertensive and diabetic patients with COVID-19 aged 18-90 years who were admitted to COVID-19 isolation wards and ICUs of Mayo Hospital Lahore were recruited in this study after obtaining informed consent and approval from the Institutional Review Board (IRB) of King Edward Medical University, Lahore (approval number: 48/RC/KEMU). Data were collected from patient charts containing age, hypertension and diabetes status, disease status, disease severity, and levels of inflammatory markers (D-dimers, serum lactate dehydrogenase (LDH), serum ferritin, and C-reactive protein (CRP)). Pre-existing diabetes and hypertension were defined by history, ongoing medications, and medical records. The primary outcome was defined based on ICU admissions and deaths. Written consent was obtained from each participant or their attendants before their involvement in the study after obtaining IRB approval.

Inclusion and exclusion criteria

COVID-19-positive patients with diagnosed hypertension and diabetes and 18-90 years of age who were admitted to COVID-19 isolation wards and ICUs of Mayo Hospital Lahore were recruited in this study after obtaining informed consent. Patients who did not provide consent, patients whose positive polymerase chain reaction reports for COVID-19 were not available, pregnant females, and patients with other comorbidities were excluded.

Sample size calculation

Following purposive selective sampling, 90 patients were included in this cross-sectional study. A sample size of 90 patients was estimated.

Statistical analysis

Data were analyzed using SPSS version 23 (IBM Corp., Armonk, NY, USA). Quantitative variables such as age were presented as mean ± SD. Qualitative variables such as hypertension, diabetes, and levels of inflammatory markers were presented as frequency and percentages.

## Results

In this study, patients admitted to COVID-19 isolation wards and ICUs were mostly between the ages of 30 and 60 years. A total of 90 patients were included, all of whom were either hypertensive, diabetic, or both. Of the 59 hypertensive patients, 46 (77.9%) were admitted to ICUs, and 13 (22%) were admitted to wards. Of the 56 diabetic patients, 44 (78.5%) patients with COVID-19 were admitted to ICUs, and 12 (21.4%) were being treated in wards.

**Figure 1:**
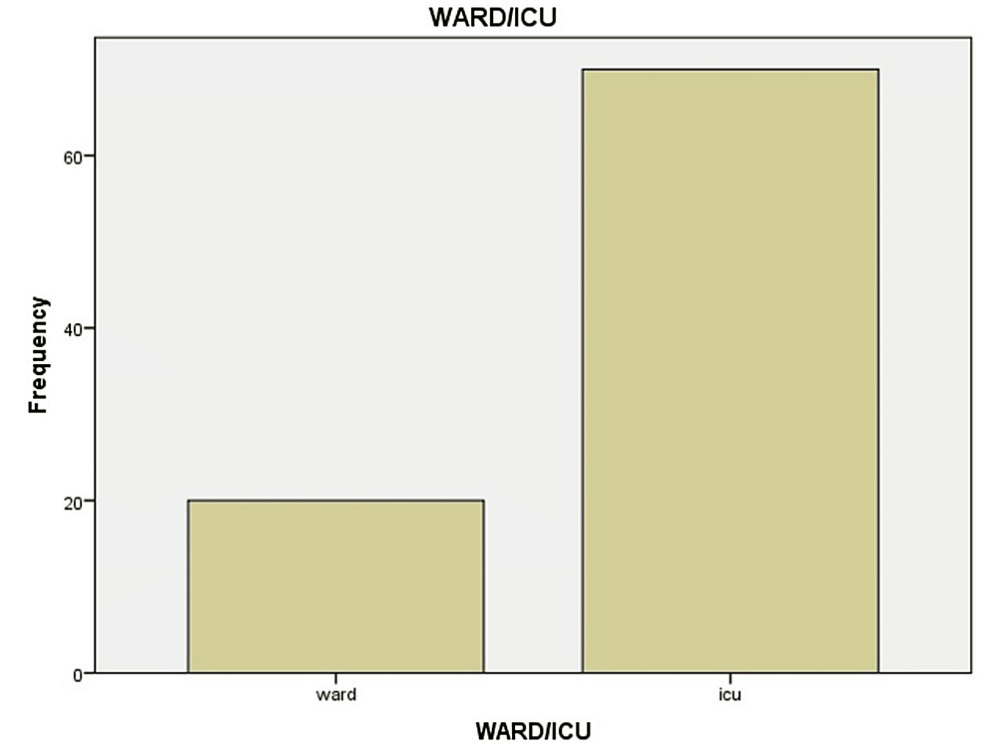
Ward/intensive care unit (ICU) admissions in diabetic and hypertensive patients showing disease severity.

**Figure 2:**
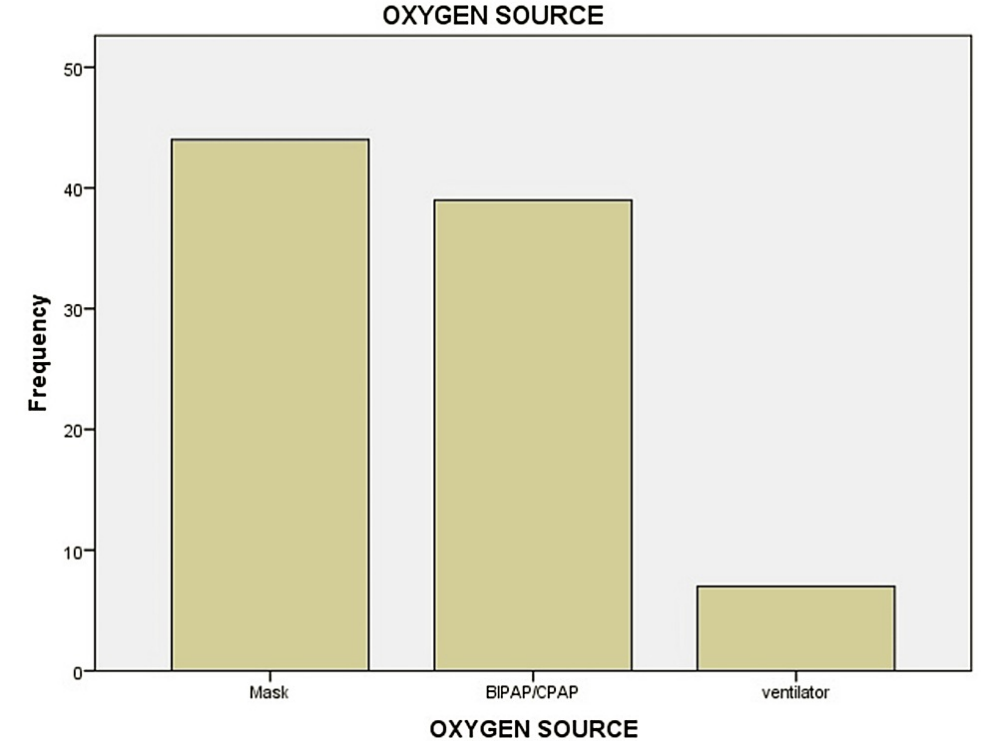
Association of diabetes with the type of oxygen source showing the severity of illness. CPAP = continuous positive airway pressure; BIPAP = bilevel positive airway pressure

Of the 90 diabetic/hypertensive patients included in the study, 70 (77.7%) were admitted to ICUs, and 20 (22.2%) were admitted to COVID-19 isolation wards (Figure 1). Among the 70 patients in ICUs, 39 (43.3%) were on CPAP/BIPAP, seven (7.8%) were on ventilators, and 44 (48.9%) were on normal oxygen masks or NRMs, mostly with high-flow oxygen (Table 1, Figure 2).

**Table 1:** Ward/ICU: oxygen source cross-tabulation. ICU = intensive care unit; CPAP = continuous positive airway pressure; BIPAP = bilevel positive airway pressure

Oxygen source		Mode of ventilation			Total
		Mask	BIPAP/CPAP	Ventilator	
Ward/ICU	Ward	20 (22.2%)	0	0	20
	ICU	24 (34.2%)	39 (43.3%)	7(7.8%)	70
Total		44	39	7	90
Percentage		48.9%	43.3%	7.8%	100%

Overall, 100% of the patients admitted to COVID-19 isolation wards and ICUs had raised levels of inflammatory markers. Of the 90 patients, 84 (93.3%) diabetic/hypertensive patients had severely high levels of CRP (>10 mg/dL), of whom 67 (79.8%) were admitted to ICUs and 17 (20.2%) to wards, and six (6.7%) patients had moderately high CRP levels (1-10 mg/dL) and were admitted to COVID-19 isolation wards and ICUs. A total of 33 (36.7%) patients had severely high levels of D-dimers (>5 µg/mL), of whom 31 (94%) were admitted to ICUs and two (6%) to wards. Overall, 57 (63.3%) patients had moderately high levels of D-dimers (0.6 to 5 µg/mL), of whom 39 (68.4%) were admitted to ICUs and 18 (31.6%) to COVID-19 isolation wards (Table 2).

**Table 2:** Ward/ICU: inflammatory markers cross-tabulation. ICU = intensive care unit; CRP = C-reactive protein; LDH = lactate dehydrogenase

Mode of admission	CRP (mg/dL)			D-dimer (µg/mL)			Serum ferritin (ng/mL)				Serum LDH (U/L)			
	1–10	>10	Total	0.6–5	>5	Total	250–500	500–1,000	>1,000	Total	250–500	500–1,000	>1,000	Total
Ward	3	17	20	18	2	20	15	3	2	20	15	3	2	20
ICU	3	67	70	39	31	70	24	23	23	70	17	34	19	70
Total	6	84	90	57	33	90	39	26	25	90	32	37	21	90

Among diabetic and hypertensive patients admitted with COVID-19, 25 (27.8%) had severely high levels of serum ferritin (>1,000 ng/mL), of whom 23 (92%) were admitted to ICUs and two (8%) to wards; 26 (28.9%) patients had moderately high levels of serum ferritin (500-1,000 ng/mL), of whom 23 (88.5%) were admitted to ICUs and three (11.5%) to wards, while 24 (61.5%) of the ICU patients and 15 (38.5%) of the ward patients had significantly raised serum ferritin levels (250-500 ng/mL). Regarding serum LDH levels, 21 (23.3%) patients had severely high levels of LDH (>1,000 U/L), of whom 19 (90.5%) were being treated in ICUs and two (9.5%) in wards. A total of 34 (92%) out of 37 (41.1%) patients with moderately high levels of LDH (500-1,000 U/L) were present in ICUs, and three (8%) were present in wards. Finally, 17 (53%) patients in ICUs and 15 (47%) patients in wards had significantly raised levels of serum LDH (250-500 U/L) (Table 2).

Of the 90 patients with hypertension or diabetes, 27 (30%) were being given antivirals. i.e., remdesivir, and 11 (12%) patients were being given an immunosuppressant, i.e., tocilizumab, while almost all of the patients were receiving steroids in the form of intravenous dexamethasone (Table 3).

**Table 3:** Effect of antivirals and immunosuppressants on mortality.

Mode of admission	Antivirals		Total	Immunosuppressants		Total	Mortality		Total
	Yes	No		Yes	No		Expired	Survived	
Ward	1	19	20	0	20	20	3	17	20
ICU	26	44	70	11	59	70	62	8	70
Total	27	63	90	11	79	90	65	25	90
Percentage	30%	70%	100%	12%	88%	100%	72.2%	27.8	100%

**Figure 3:**
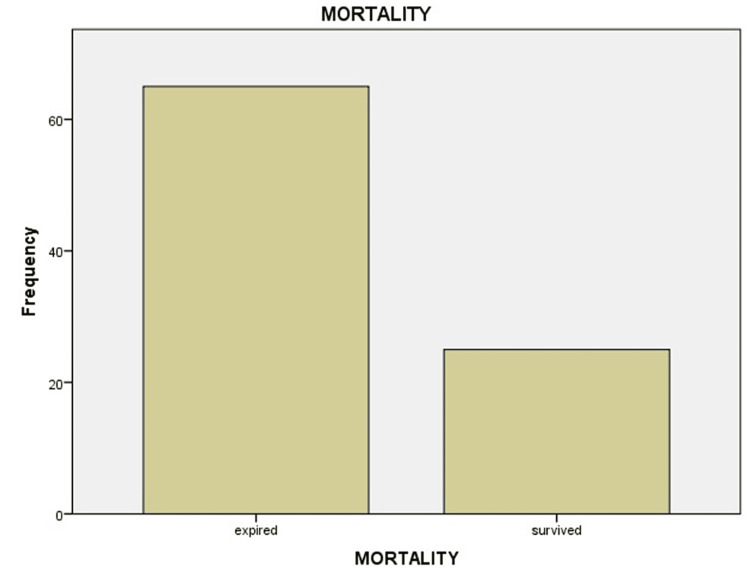
Mortality in diabetic and hypertensive patients.

Among the 90 patients, 65 (72.2%) expired and 25 (27.8%) survived (Figure 3).

Of the expired patients, 62 (95.3%) were admitted to ICUs, and three (4.6%) were admitted to wards. The mortality in our study was found to be associated with diabetes (odds ratio (OR) = 1.2; 95% confidence interval (CI) = 1.28-1.48) and hypertension (OR = 1.1; 95% CI = 1.24-1.44).

## Discussion

This recent breakout of the novel COVID-19, as well as its rapid global expansion, poses a global health threat [7]. However, the factors that influence severity and mortality have not been well investigated. Identifying major risk factors and applying appropriate treatment measures can help save many lives. The majority of prior research has identified comorbidities such as hypertension and diabetes mellitus as high-risk factors and provided information on the implications of these pre-existing diseases on the severity of COVID-19 for physicians to identify the main risk factors for a severe outcome of the disease [8]. Only in-patients were included in our study. The following findings may be obtained by focusing on the comorbidities linked with COVID-19 severity and utilizing in-hospital all-cause death as an endpoint.

First, having a prior diagnosis of hypertension raised the risk of mortality in COVID-19 patients who needed hospitalization by almost 20%, regardless of age or other cardiovascular comorbidities. Our findings corroborate the fact that old adults are more likely to exhibit worse illness presentations with COVID-19-positive tests and require hospital admission compared to younger individuals, which is supported by a great majority of investigations [9,10].

In our study, we found a strong positive association between diabetes mellitus, hypertension, and COVID-19 severity and mortality. Our results suggest an overall prevalence of diabetes at 78% and hypertension at 78.5% for ICU admissions. Our results also show a mortality of 72%, highlighting that these comorbidities are associated with an increased risk for ICU admission and mortality. The mortality in our study was found to be associated with diabetes (OR = 1.2; 95% CI = 1.28-1.48) and hypertension (OR = 1.1; 95% CI = 1.24-1.44).

Hypertension was shown to be prevalent in 26% to 30% of COVID-19 patients in early reports [10]. Pranata et al. [11] conducted a meta-analysis involving 6,560 participants from 30 papers published in PubMed and other databases to identify a connection between hypertension and COVID-19 severity and mortality. Hypertension was linked to an increased risk of death (relative risk (RR) = 2.21 (1.74, 2.81), p < 0.001) and COVID-19 severity (RR = 2.04 (1.69, 2.47), p < 0.001) [11].

Kumar et al. [12] found an OR of 2.75 (95% CI = 2.09 to 3.62) for the relationship between diabetes and severity and an OR of 1.90 (95% CI = 1.37 to 2.64) for diabetes and mortality [12]. Another recent meta-analysis that included approximately 30 studies (16,003 and 6,452 patients, respectively) aimed to investigate the relationship between solely diabetes and COVID-19 severity and mortality. According to the research, diabetes was linked to mortality (RR = 2.12 (1.44, 3.11), p 0.001; I^2^ = 72%) and the severity of COVID-19 infection (RR = 2.45 (1.79, 3.35), p 0.001; I^2^ = 45%) [13].

The association between hypertension, diabetes, and worse COVID-19 infection outcomes might be attributable to the greater prevalence of comorbidities and the individuals’ older age. Hypertension was not shown to be an independent factor influencing COVID-19 outcomes in an Italian cross-sectional investigation [14].

This study has several limitations. The effects of diabetes and hypertension cannot be attributed only to these exposures as individuals may have had other comorbidities such as renal disease, coronary heart disease, or respiratory distress syndrome as well. Second, the majority of the investigations were conducted entirely in China. Additional data will become accessible as the disease expands throughout the world, allowing researchers to see if comparable conclusions apply to other groups. Third, multiple disorders typically coexist in the same people, and it is possible that one of them increases the risk assigned to the others. Finally, we were unable to analyze the interaction of age, obesity, glycemic control, and type of diabetes because of the limited literature available [15,16].

Nonetheless, our study has notable strengths as well, including a large number of participants, the inclusion of certain demographic and biochemical values such as age, blood pressure, glycemic record, duration in hospital, and several biochemical inflammatory markers such as CRP, D-dimers, LDH, and serum ferritin. Taking all of these factors into account, our findings support the relevance of diabetes and hypertension to COVID-19 severity and mortality.

This data would be useful not just for future systematic studies but also for frontline doctors to identify the COVID-19 risks that their patients face. Patient characteristics, subtypes of hypertension or diabetes, length of disease, drugs taken, and disease control indicators should all be reported in future studies of COVID-19 patients with diabetes or hypertension [3]. Because of the wide range of definitions of COVID-19 severity, we advocate utilizing the ICU admission rate as a more objective approach to determining severity until a uniform definition of COVID-19 severity is accepted.

Chronic disorders such as diabetes and hypertension have been linked to a poor prognosis in studies [17,18]. To combat the expanding burden of the COVID-19 pandemic, it is required to improve medical certification of causes of death across nations as well as develop an analytical process for standard minimum data reporting [19].

## Conclusions

Our results revealed that diabetes and hypertension are strongly associated with poor disease outcomes for COVID-19. Hence, these diseases are strong predictors of COVID-19 severity and are associated with higher risk in terms of morbidity and mortality due to respiratory deterioration. The notable strengths of our study include the thorough clinical characterization of patients with the use of several biochemical inflammatory markers. Patients with these diseases face a higher risk of poor outcomes compared with those without these comorbidities. This also markedly reinforces the clinical message that diabetes and hypertension are significant prognostic indicators for unfavorable outcomes during COVID-19. Hence, these two are the objectives that must be aggressively tackled in the management of COVID-19.

## References

[1] Muniyappa R, Gubbi S (2020). COVID-19 pandemic, coronaviruses, and diabetes mellitus. Am J Physiol Endocrinol Metab.

[2] Tadic M, Cuspidi C (2021). The influence of diabetes and hypertension on outcome in COVID-19 patients: do we mix apples and oranges?. J Clin Hypertens (Greenwich).

[3] Barrera FJ, Shekhar S, Wurth R (2020). Prevalence of diabetes and hypertension and their associated risks for poor outcomes in Covid-19 patients. J Endocr Soc.

[4] Fadini GP, Morieri ML, Boscari F (2020). Newly-diagnosed diabetes and admission hyperglycemia predict COVID-19 severity by aggravating respiratory deterioration. Diabetes Res Clin Pract.

[5] Sun Y, Guan X, Jia L (2021). Independent and combined effects of hypertension and diabetes on clinical outcomes in patients with COVID-19: a retrospective cohort study of Huoshen Mountain Hospital and Guanggu Fangcang Shelter Hospital. J Clin Hypertens (Greenwich).

[6] Ferrannini E, Cushman WC (2012). Diabetes and hypertension: the bad companions. Lancet.

[7] Zaki N, Alashwal H, Ibrahim S (2020). Association of hypertension, diabetes, stroke, cancer, kidney disease, and high-cholesterol with COVID-19 disease severity and fatality: a systematic review. Diabetes Metab Syndr.

[8] Zhou F, Yu T, Du R (2020). Clinical course and risk factors for mortality of adult inpatients with COVID-19 in Wuhan, China: a retrospective cohort study. Lancet.

[9] Guan WJ, Ni ZY, Hu Y (2020). Clinical characteristics of coronavirus disease 2019 in China. N Engl J Med.

[10] Guan WJ, Liang WH, Zhao Y (2020). Comorbidity and its impact on 1590 patients with COVID-19 in China: a nationwide analysis. Eur Respir J.

[11] Pranata R, Lim MA, Huang I, Raharjo SB, Lukito AA (2020). Hypertension is associated with increased mortality and severity of disease in COVID-19 pneumonia: a systematic review, meta-analysis and meta-regression. J Renin Angiotensin Aldosterone Syst.

[12] Kumar A, Arora A, Sharma P (2020). Is diabetes mellitus associated with mortality and severity of COVID-19? A meta-analysis. Diabetes Metab Syndr.

[13] Huang I, Lim MA, Pranata R (2020). Diabetes mellitus is associated with increased mortality and severity of disease in COVID-19 pneumonia - a systematic review, meta-analysis, and meta-regression. Diabetes Metab Syndr.

[14] Iaccarino G, Grassi G, Borghi C, Ferri C, Salvetti M, Volpe M (2020). Age and multimorbidity predict death among COVID-19 patients: results of the SARS-RAS study of the Italian Society of Hypertension. Hypertension.

[15] Zhang X, Yu J, Pan LY, Jiang HY (2020). ACEI/ARB use and risk of infection or severity or mortality of COVID-19: a systematic review and meta-analysis. Pharmacol Res.

[16] Cai Q, Chen F, Wang T (2020). Obesity and COVID-19 severity in a designated hospital in Shenzhen, China. Diabetes Care.

[17] Lu Q, Shi Y (2020). Coronavirus disease (COVID-19) and neonate: what neonatologist need to know. J Med Virol.

[18] Richardson S, Hirsch JS, Narasimhan M (2020). Presenting characteristics, comorbidities, and outcomes among 5700 patients hospitalized with COVID-19 in the New York City area. JAMA.

[19] Rao C (2020). Medical certification of cause of death for COVID-19. Bull World Health Organ.

